# Development and evaluation of a machine learning prediction model for short-term mortality in patients with diabetes or hyperglycemia at emergency department admission

**DOI:** 10.1186/s12933-025-02954-8

**Published:** 2025-10-03

**Authors:** Per Wändell, Marcelina Wierzbicka, Karolina Sigurdsson, Anna Olofsson, Caroline Wachtler, Torgny Wessman, Olle Melander, Ulf Ekelund, Anders Björkelund, Axel C. Carlsson, Toralph Ruge

**Affiliations:** 1 Department of Neurobiology, Care Sciences and Society, Division of Family Medicine and Primary Care, Karolinska Institutet, Huddinge, Sweden; 2 Center for Primary Health Care Research, Lund University, Malmö, Sweden; 3 Department of Emergency and Internal Medicine, Skånes University Hospital, Malmö, Sweden; 4 Department of Clinical Sciences Malmö, Department of Internal Medicine, Lund University, Skåne, Sweden; 5 Division of Biostatistics, Institute of Environmental Medicine, Karolinska Institutet, Stockholm, Sweden; 6 Academic Primary Health Care Centre, Region Stockholm, Stockholm, Sweden; 7 School of Health and Social Studies, Dalarna University, Falun, Sweden; 8 Emergency medicine, Department of Clinical Sciences Lund, Department of Emergency Medicine, Skåne University Hospital, Lund University, Lund, Sweden; 9 Centre for Environmental and Climate Science, Lund University, Lund, Sweden

**Keywords:** Artificial intelligence, Diabetes, Emergency medicine, Gradient boosting, Normalized relative influence, Prediction

## Abstract

**Background:**

Patients with diabetes admitted to emergency care face a higher risk of complications, including prolonged hospital stays, admissions to the intensive care unit and mortality.

**Aim:**

To develop a machine learning (ML) model to predict 30-day mortality in patients with diabetes admitted to the emergency department (ED).

**Design and setting:**

A cohort study utilizing data from all nine ED’s in Region Skåne 2017 to 2018. Totally 74,611 patient visits, representing 34,280 unique patients aged > 18 years with diabetes or hyperglycemia (glucose were > 11 mmol/L). The analysis focused on four groups, men and women aged 40–69 and ≥ 70 years.

**Methods:**

Stochastic gradient boosting was employed to develop a model predicting 30-day mortality. Variable importance was assessed using normalized relative influence (NRI) scores. Variables in certain hospitals were used to train the models, and the models were tested in other hospitals.

**Results:**

Key predictors included laboratory values (pH, base excess, pCO_2_, standard bicarbonate, oxygen saturation, lactate, CRP, and leukocytes), as well as age, triage category, and time to doctor consultation. The sensitivity of the models ranged from 86–97%, the specificity from 86–94%, and accuracy between 86% and 94%. The area under the curve (AUC) ranged from 0.84 to 0.93 and Cohen’s kappa ranged from 0.34 to 0.45. Positive predictive values accurately identified mortality in 23% to 37% of cases across the four groups.

**Conclusions:**

A machine learning model based on routinely collected data in the ED accurately predicted 30-day mortality with high specificity and sensitivity. This approach shows promise in identifying high-risk patients requiring close monitoring and timely interventions.

**Supplementary Information:**

The online version contains supplementary material available at 10.1186/s12933-025-02954-8.

## Introduction

Diabetes is a chronic disease that has become a global epidemic [1]. Mortality among individuals with diabetes is estimated to be two to four times higher compared to those without the condition, and diabetes is the seventh leading cause of death in the United States in 2019 [2]. People with diabetes are up to four times more likely to require hospitalization compared to individuals without diabetes [3–6]. Data indicates that 30% of patients discharged with a diabetes diagnosis will require two or more hospitalizations within a single year [5, 7, 8].

In Sweden, the prevalence of diabetes is estimated to around 5% [9]. The excess risk of ischemic heart disease [10], heart failure [11], and mortality [12], in individuals with diabetes compared to those without, is highest in the age < 55 years of age, and decreases to a fairly equal risk at ≥ 75 years of age.

Diabetes increases a person’s risk for visiting the emergency department [13], and some of these individuals have increased risk of adverse outcomes like mortality, following an ED visit. Additionally, those hospitalized with a diabetes diagnosis tend to have longer hospital stays than those without diabetes, even when admitted for the same medical conditions [7, 14, 15].

The use of machine learning (ML) for outcome prediction, including mortality, has shown promise in ED settings. ML systems are capable of detecting patterns in large databases using programmed algorithms, which can provide clinical benefits by predicting outcomes from health records, analyzing various medical conditions, and aiding in diagnosing chronic diseases and assessing their severity [16]. This approach is particularly advantageous because ML can identify relationships between key patient predictors and various outcomes across many clinical processes, ultimately assisting healthcare professionals in decision-making. ML applications that incorporate triage systems [17], blood sampling, vital signs and length of stay have been promising in predicting mortality, the need of critical care, complications and length of hospital stay [18–20]. However, to our knowledge, no such ML method has been specifically applied to patients with diabetes.

There is a significant negative impact of diabetes and hyperglycemia on clinical outcomes for critically ill patients, including those admitted to intensive care units. The aim of this study was to develop and evaluate a machine learning (ML) prediction model using all ED visits in Region Skåne, Sweden between 2017 and 2018, to predict 30-day mortality and non-mortality in patients with diabetes admitted to the ED.

## Methods

### Study design, data sources, participants and predictors

This retrospective study used a cohort design. Data for the development and evaluation data sets were collected from nine EDs in the Region Skåne catchment area (Malmö, Lund, Helsingborg, Ystad, Ängelholm and Trelleborg) between 2017 and 2018. The cohort consisted of approximately 632,744 patient visits, representing 325,539 unique patients aged > 18 years.

Individuals with diabetes were identified, based on the following criteria:

- A diagnosis coded according to ICD-10 within 12 months prior to the ED admission,
- A prescription of antidiabetic medications within 12 months prior to the ED admission, or
- A random non-fasting blood glucose measurement greater than 11 mmol/L taken at the ED.

The primary outcome for prediction in this study was 30-day mortality following admission to the ED as an easily detected outcome in individuals fitting this wide diabetes definition. To make the results applicable for clinicians with patients admitted to the ED most relevant, all patients fitting our definitions were included. Both those later admitted and those discharged home.

### Studied features in machine learning models

The study cohort is drawn from Region Skåne clinical data with detailed information on care processes. Variables for all patients originate from the regional patient administrative system (PAS) and include:

- Number of healthcare contacts, by contact type.
- Laboratory tests, including blood tests and microbiological analyses.
- Physiological examinations such as ECG, lung function tests, and urine measurements.
- Surgical procedures.
- Referrals to other healthcare providers.
- Medication treatments.

#### Index emergency department (ED) visit

Collected variables comprise presenting complaint, mode of arrival, triage category, time stamps (time to physician and total ED length of stay); physical examination findings such as blood pressure, oxygen saturation, pulse, auscultation; investigations within 24 h of arrival with results blood tests, cultures and microbiology, diagnostic imaging, ECG; disposition (admitted/discharged, left against medical advice), and the ED physician’s working hypothesis/preliminary diagnosis or initial assessment.

Post-ED data include inpatient length of stay, intensive care during and within 30 days of the index visit, outpatient and inpatient diagnoses and procedure codes including: critical interventions such as defibrillation for cardiac arrest, thrombolysis for stroke, and emergent PCI, ED revisits, hospital admissions and number of inpatient days, date of death, and registered causes of death.

Frailty was defined using the National Board of Health criteria, based on the number of hospital and primary care admissions, the number of registered chronic diseases, and the number of prescribed medications [21].

The data from the ED were linked to national registers. To the National Patient Register to obtain ICD-10 codes of common comorbidities registered before the ED visit; to the National Prescribed Drug Register to obtain prescribed medications collected from pharmacies; as well as the National Cancer Register and the National Register of Death. ), Finally, the Central Bureau of Statistics in Sweden added information about the patients’ socioeconomic status and country of birth.

### Ethics

Ethical approval was obtained to use the clinical data and to link them to national registers without patient consent.

### Analytical methods

Stochastic gradient boosting (SGB) [22] is a modern machine learning method used to predict clinical outcomes. It has been used previously by us to differentiate care seeking patterns prior a diagnosis in case-control designs [23, 24]. Here, we used a cohort design to predict mortality. All visits from EDs in Malmö, Helsingborg, Ystad and Trelleborg were used to train the SGB models and visits in Lund, Kristianstad and Hässleholm were used to test the SGB model performance in an independent cohort, with the same type of patients in a similar setting.

Hyperparameters were chosen to balance flexibility and regularization. A large maximum number of trees (20,000) was combined with a small learning rate (0.001), so that boosting proceeded in many small steps with the effective number of trees determined by 10-fold cross-validation (early stopping). Tree depth was fixed at 5 to allow moderate interactions without excessive complexity. Additional regularization included subsampling (bag fraction = 0.5) and a minimum of 10 observations per terminal node. These settings follow common practice for clinical prediction modeling with gradient boosting and were selected to minimize overfitting while maintaining adequate predictive capacity.

Once the model was trained, it was applied to both training and test datasets to predict individual probabilities of the outcome–in this case, 30-day mortality for each patient. We established cut-off values for classification by calculating the percentile of individual probabilities from the training set that corresponded to the proportion of patients without the outcome. In the test set, a patient was classified as having the outcome (30-day mortality) if their individual probability from the SGB model exceeded this cut-off value; otherwise, the patient was classified as not having the outcome. The performance of the SGB models was evaluated using area under the receiver operator characteristic (ROC) curve (AUC), confusion matrices, overall accuracy, sensitivity, specificity, positive predicted value (PPV), and negative predicted value (NPV) [22].

Calibration of the SGB models was evaluated by calculating the Brier score and by constructing calibration plots, see Supplementary Figs. 1–4. Predicted probabilities from the models were grouped into quantiles, and within each group the mean predicted probability was compared with the observed event rate. Confidence intervals for observed event rates were estimated using binomial standard errors.

To assess potential overfitting, we compared model discrimination in the training and independent test cohorts. All models were trained with a large maximum number of trees, and the effective number of iterations for evaluation was selected using 10-fold cross-validation (early stopping). Area under the ROC curve (AUC) was then calculated for both training and test sets, and differences between the two were used as an indication of model optimism.

Variable importance was assessed using normalized relative influence (NRI) scores, where the relative influence values were normalized to sum to 100 [25].

## Results

**Fig. 1:**
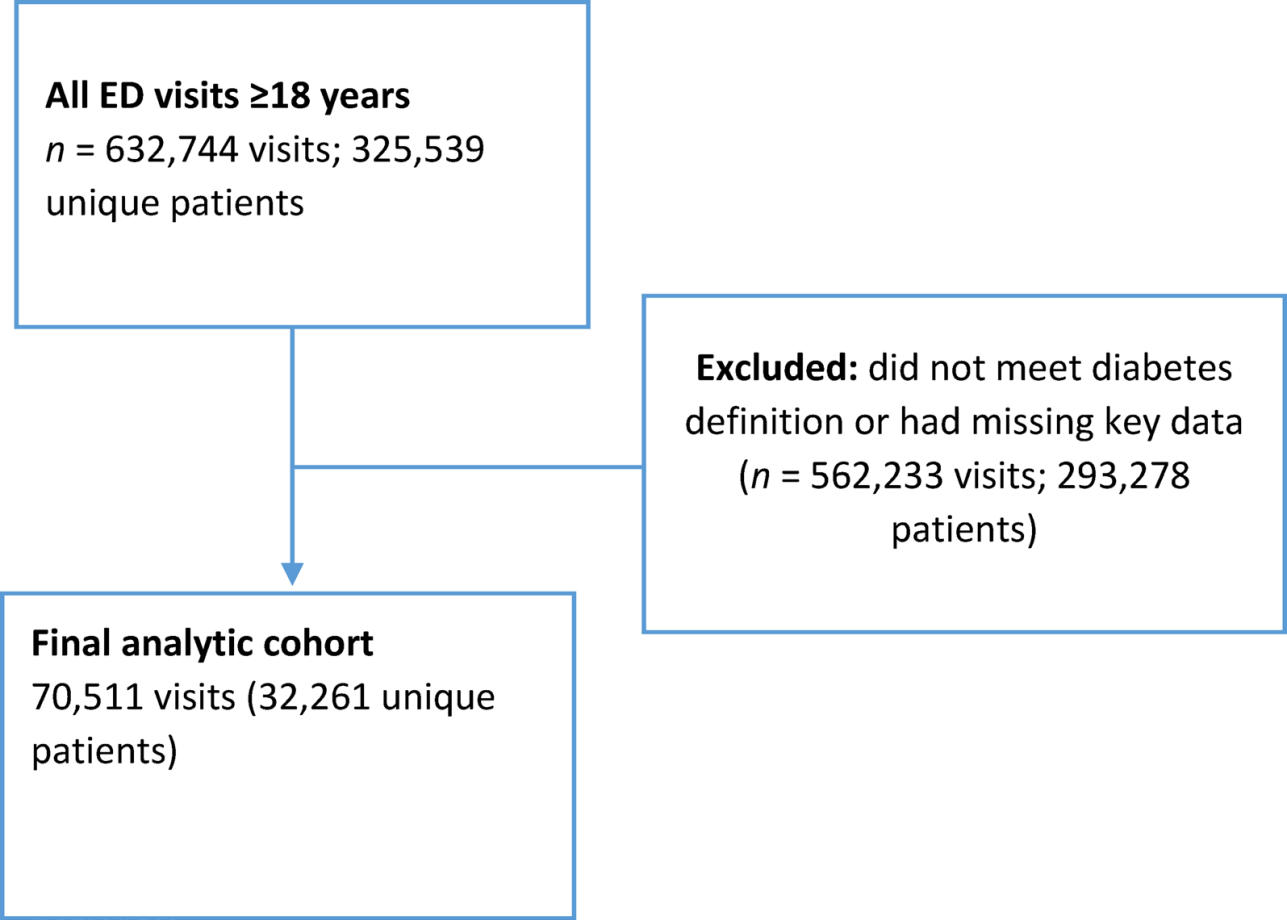
Study flow diagram. Diabetes definition: ICD-10 code within 12 months prior to the ED admission, or antidiabetic prescription within 12 months prior to the ED admission, or random non-fasting blood glucose > 11 mmol/L measured at the ED

**Fig. 2:**
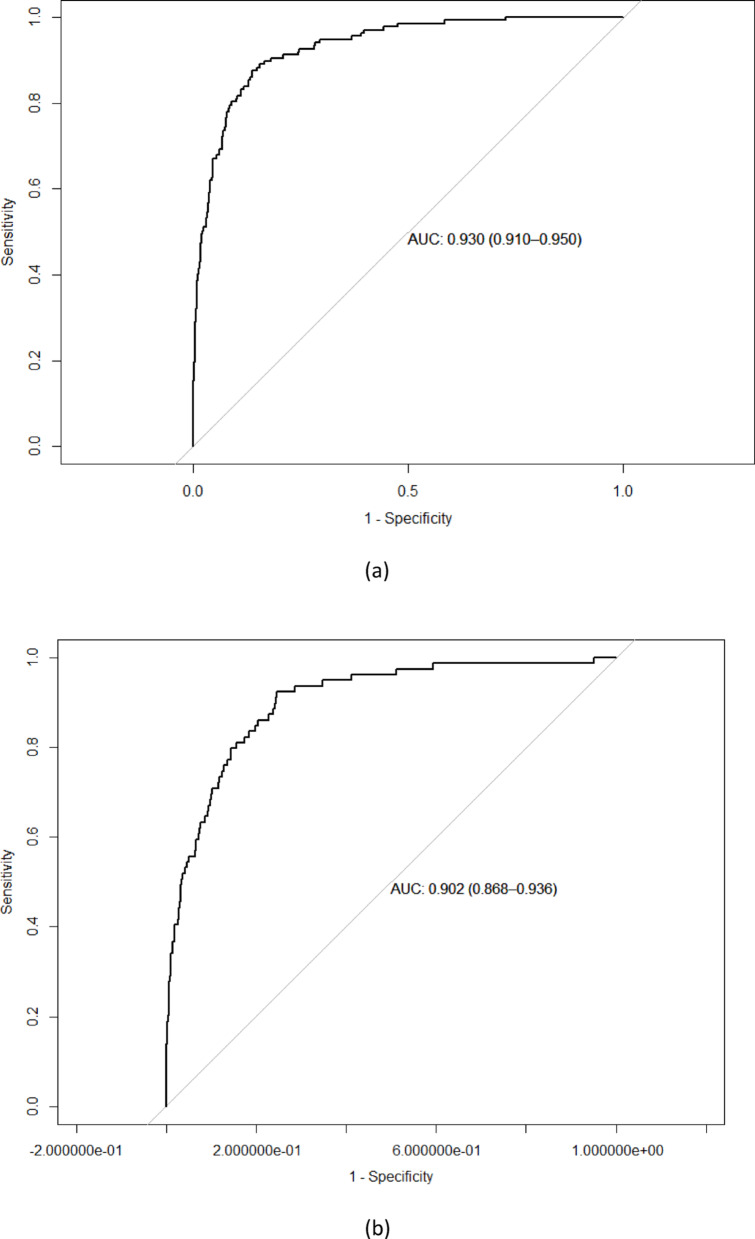

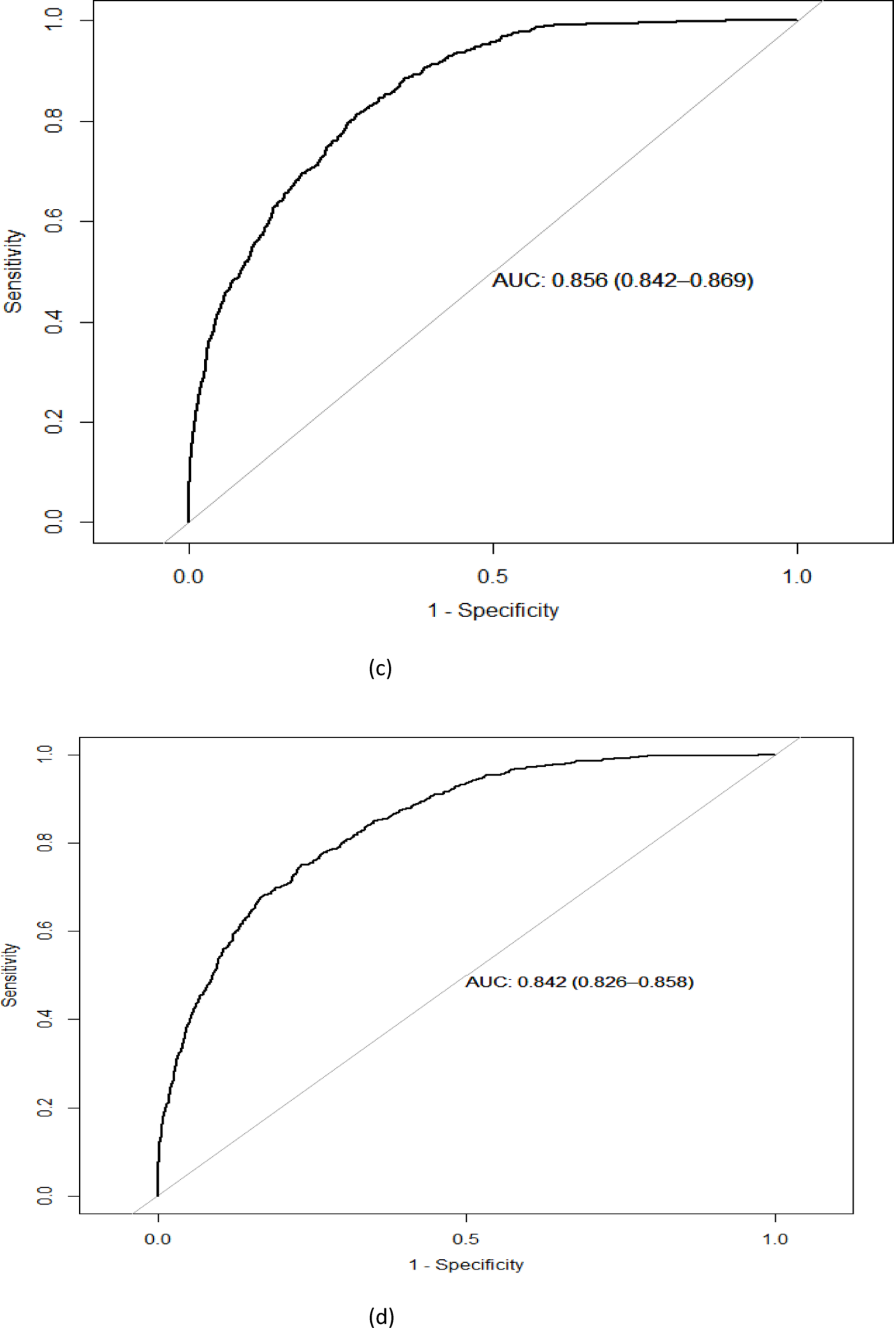
**a**. Receiver operating curves for the machine learning models in the test data set for men 40 to 69 years of age.** b**. Receiver operating curves for the machine learning models in the test data set for women 40 to 69 years of age. **c**. Receiver operating curves for the machine learning models in the test data set for men ≥ 70 years of age. **d**. Receiver operating curves for the machine learning models in the test data set for women ≥ 70 years of age

The study cohort included 70,511 observations, representing 32,261 unique patients, as shown in Fig. 1. The observed vs. predicted values for 30-day mortality and non-mortality are shown in Table 1. The model’s ability to predict non-mortality was high across all groups (Supplementary Table S1), i.e. the negative predictive value (NPV) was 99.7%, whereas the positive predictive value (PPV) was 30%. The predicted risk of 30-day mortality was consistently lower than the observed risk (Table 2). The receiver operating curves are shown as Fig. 2a, b, c and d.

**Table 1:** Observed vs. predicted 30-day mortality for patients with diabetes seeking on emergency departments in region Skåne, Sweden in the test data set

Predicted	Observed		
	No death 30 days	Death_30days	Total
Men 40–69 y			
No death_30days	8397 (99.7%)	22 (0.3%)	8419
Death_30days	546 (69.6%)	239 (30.4%)	785
Total	8943 (97.2%)	261 (2.8%)	9204
Men ≥ 70 y			
No death_30days	9006 (98.5%)	141 (1.5%)	9147
Death_30days	1437 (63.1%)	840 (36.9%)	2277
Total	10,443 (91.4%)	981 (8.6%)	11,424
Women 40–69 y			
No death_30days	5520 (99.9%)	4 (0.1%)	5524
Death_30days	523 (77.0%)	156 (23.0%)	679
Total	6043 (97.4%)	160 (2.6%)	6203
Women ≥ 70 y			
No death_30days	7545 (98.5%)	113 (1.5%)	7658
Death_30days	1176 (62.6%)	703 (37.4%)	1879
Total	8721 (91.4%)	816 (8.6%)	9537

**Table 2:** Statistical performance of the models in the test data sets for the four sex- and age-groups

Group	Sensitivity	Specificity	Accuracy	Cohen’s Kappa	AUC (95% CI)
Men 40–69 y	0.92	0.94	94%	0.43	0.980 (0.972–0.987)
Men ≥ 70 y	0.86	0.86	86%	0.45	0.939 (0.932–0.947)
Women 40–69 y	0.97	0.91	92%	0.34	0.978 (0.964–0.992)
Women ≥ 70 y	0.86	0.87	86%	0.46	0.939 (0.931–0.947)

### Men

#### Men aged 40–69 years

*For men aged 40–69 years*, the optimal number of trees for the model was 11,289. The model demonstrated a sensitivity of 92% and a specificity of 94% (Table 2). The accuracy, defined as the proportion of correct predictions, was 94%. Cohen’s Kappa, which measures agreement between the model’s predictions and actual outcomes, was 0,43, indicating moderate agreement. In summary, the model displayed a high sensitivity, meaning it is effective at identifying true positive cases (i.e. patients who did die within 30 days). However, the Positive Predictive Value (PPV) was lower, around 30%, suggesting that while the model predicted mortality accurately for some cases, 70% of the positive predictions were false positives.

#### Men aged ≥ 70 years

Among men ≥ 70 years, the optimal number of trees was 19,994. The sensitivity and specificity were both 86%, (Table 2). The accuracy was 86%, with a Cohen’s Kappa of 0,45, suggesting a moderate agreement between predicted and actual outcomes. The PPV for this group was 37%, which, while higher than for the younger group, still indicated that 63% of the predicted positive cases (i.e. mortality within 30 days) were false positives.

#### Key predictive variables for men

The top 10 variables for predicting mortality are listed in Table 3, categorized by both age and sex (men and women aged groups of 40–69 years and ≥ 70 years), and as Supplementary Figs. 5–8. Most of these variables were laboratory test results, in detail measurements that provide information about infection and ketoacidosis. Other important predictors included age, time to doctor consultation, and triage category.

**Table 3:** Top 10 variables associated with death within 30 days in men and their normalized relative influence (NRI)

Group	Variable	NRI (%)
Men 40–69 y		
	Venous Blood-pH	9.96
	Blood-Ecv Base excess	9.46
	Venous Blood-pCO_2_	8.58
	Plasma-Standard bicarbonate	8.16
	Venous Blood-Oxygen saturation	5.45
	Plasma-Lactate	5.41
	Plasma-Crp	4.86
	Age	3.95
	Time to doctor consultation	3.76
	Blood-Leukocytes	3.69
Men ≥ 70 y		
	Plasma-Crp	7.67
	Blood-Ecv Base excess	7.61
	Age	7.00
	Plasma-Lactate	6.37
	Plasma -Natrium	5.98
	Venous Blood-pCO_2_	5.13
	Triage category	4.87
	Plasma-Standard bicarbonate	4.11
	Venous Blood-pH	4.01
	Blood-Leukocytes	3.97

Crp: C-reactive protein

Ecv: extracellular volumes

pCO_2_: pressure of carbon dioxide

### Women

#### Women aged 40–69 years

For women *aged* 40–69 years, the optimal number of trees was 7145. The model demonstrated a sensitivity of 97%, specificity of 91%, and an accuracy of 92%. along with a Cohen’s Kappa of 0.34. While the model showed a high sensitivity, indicating it was effective at identifying true positive cases, the Positive Predictive Value (PPV) was lower. When the model predicted a positive outcome (i.e., mortality within 30 days), it was correct 23% of the time.

#### Women aged ≥ 70 years

Among women aged  ≥ 70 years, the optimal number of trees was 16,584. The model achieved a sensitivity of 86%, specificity of 87%, and an accuracy of 86%. Cohen’s Kappa was 0.46, reflecting moderate agreement. While the model exhibited relatively high sensitivity, the PPV was 37%, meaning that when predicting mortality, it was correct in 37% cases.

#### Key predictive variables for women

Overall, the models for women demonstrated high sensitivity and specificity, along with moderately high accuracy. However, the PPVs were relatively low, suggesting that many of the positive predictions were false positives. Depending on the specific goals of the analysis, further fine-tuning of the model could be beneficial to improve precision or meet other specific performance criteria.

The top 10 variables for predicting mortality in women are shown in Table 4, divided into women aged 40–69 years, and women aged ≥ 70 years, respectively. As among men, most of the key variables were laboratory tests, alongside factors such as age, time to doctor consultation, and triage category.

**Table 4:** Top 10 variables associated with death within 30 days in women within 30 days and their normalized relative influence (NRI)

Group	Variable	NRI (%)
Women 40–69 y		
	Blood-Ecv-Base excess	12.27
	Plasma-Lactate	10.37
	Venous Blood-pH	7.01
	Plasma-Standard bicarbonate	6.40
	Plasma-Crp	5.92
	Triage category	4.92
	Venous Blood-pCO_2_	4.80
	Time to doctor consultation	4.11
	Plasma-Ca^++^	3.69
	Blood-glucose	3.38
Women ≥ 70 y		
	Blood-Ecv-Base excess	9.51
	Plasma-Crp	7.76
	Plasma-Lactate	6.75
	Plasma-Natrium	6.47
	Age	5.21
	Plasma-Standard bicarbonate	4.95
	Blood-Leukocytes	4.57
	Venous Blood-pH	4.05
	Plasma-Potassium	3.89
	Triage category	3.68

Crp: C-reactive protein

Ecv: extracellular volumes

pCO_2_: pressure of carbon dioxide

## Discussion

### Main findings

In this study, we developed and evaluated a prediction model for 30-day mortality in patients with diabetes admitted to the emergency department (ED), using a machine learning (ML) method. The model performed well in identifying patients with a low risk of mortality, but although it was sensitive in detecting patients at high risk of dying, the positive predictive value was lower. This means the model overpredicted deaths and classified many patients as high-risk, even though they survived. However, the NPV was high, meaning that with good safety, low risk patients could be identified, and a future use of the tool could possibly be to discharge patients from the ED. One of our key findings was that the specific combinations of blood gas values–primarily pH, lactate, base excess, bicarbonate—were predictors of mortality. In addition to these laboratory parameters, other variables associated with increased mortality included C-reactive protein (CRP), age, triage category, sodium and potassium levels in blood, and length of stay in the ED.

As in the current study, previous ML modeling studies conducted in emergency medicine have shown ML can improve risk prediction in acutely ill patients using inexpensive and easily available variables, such as vital parameters and laboratory tests collected upon ED admission [19, 26]. As in the current study, triage variables such as age, blood pressure, pulse rate and respiratory rate, oxygen saturation, and whether an ambulance was used for transport have been shown to have high predictive power [16].

Prediction models built using ML have been developed to predict various outcomes, such as length of stay [22] and mortality [20, 22, 25–29]. Predictive variables identified in these models include lumbar disc displacement [22], age [22, 25, 28], hearing loss [22], infections [22], vascular diseases [22], physiological scores [26, 28], urinary output [28], treatment with statins [29], and laboratory tests including albumin [25] (26), lactate [25, 28], blood urea nitrogen, BNP and blood neutrophile-to-lymphocyte ratio [27], as well as NT-proBNP [29]. Some of these models have demonstrated significant predictive power, with AUC of 0.95 [26] and 0.97 [20]. The use of ML to predict outcome in patients with diabetes has been explored in emergency medicine settings, but studies remain sparse and typically focuses on in-hospital patients or critically ill patients [28].

Our findings are consistent with previous research, showing that age, length of stay in the ED, abnormal test results regarding electrolyte balance, acidosis, or infection collected during triage are all important for predicting patient outcomes. Our data confirms previous observations that longer lengths of stay in the emergency department are associated with unfavorable outcomes and complications in acutely ill patients [29]. For example, an English cross-sectional, retrospective observational study demonstrated that delays in hospital admission beyond five 5 h arrival at the ED significantly increase all-cause 30-day mortality [30]. For every 82 patients with admission delays of 6 to 8 h from time of arrival at the ED, there is one additional death [30]. This effect is particularly harmful for elderly patients [29]. Also, abnormal blood test results, such as markers for sodium balance and infections, have been associated with negative clinical outcomes in patients both with and without diabetes [18–20, 31, 32]. Diabetes and hyperglycemia lead to dysfunction of the immune system, increasing the risk of severe infections, sepsis, organ failure and death [33]. The plasma lactate level indicates tissue hypoxia, with strong association between elevated lactate levels and tissue oxygen deficiency [34]. Lactate measurements are increasingly used as a prognostic tool, as it can indicate cellular hypoxia and compromised tissue circulation that can lead to metabolic acidosis, particularly in conditions like sepsis and shock [34]. Other blood gas parameters such as base excess and pH also predict mortality in critically ill patients [35]. A Danish study [36] found that higher lactate levels, lower pH, and abnormal CO_2_ levels were linked to increased 7-day and 30-day mortality, as well as a higher risk of ICU admission. A retrospective study of 274 patients admitted from the ED to the ICU, showed that lactate, base excess, and bicarbonate levels were strongly associated with mortality [37]. There was a 62% mortality rate in patients with baseline base excess values of − 2 mmol/L or, supporting our present findings that lactate is a significant predictor of mortality [37], as base excess and lactate levels are correlated with each other [38].

There were differences in the prediction of mortality in women and men in the present study, which is interesting but could be attributed to the different underlying conditions and differences in care seeking behavior. In a recent study of the top 30 diagnoses in primary care, only three were more common among men; atrial fibrillation, coronary heart disease and type 2 diabetes [39], which may explain differences in data that enters the machine learning models.

## Strengths and limitations

Our study successfully applied SGB models to predict 30-day mortality risk in patients with diabetes admitted to emergency departments. A key strength of the study is the model’s high sensitivity and specificity, indicating that it performs well in identifying patients with both low and high risk of mortality. Another strength was that we trained the model at a few EDs and tested the SGB model in other EDs showing that the model works in other hospitals than we used in the training data set. An overall strength with tree-based machine learning models is that variables with missing data can be used without imputations or exclusions, which is the case in regression modelling. One possible limitation concerns the uncertainty about the quality in the data in the study, yet the high AUC suggests that the data has sufficient quality to be used in prediction models.

## Clinical implications

For practical use in EDs, the model could rule out cases with a low mortality risk with high accuracy, making the models suitable to rule out patients who may not require intensive care, or who don’t need intensified monitoring and possibly discharge from the ED. Our model demonstrated a high degree of accuracy in predicting individuals with a low probability of mortality within 30 days. However, further refinement of the model could be applied to reduce the incidence of false positives, which currently may limit its ability to precisely identify high-risk patients. This could be done by developing the model in another, larger population. Improving the model’s positive predictive value would enhance its utility in accurately identifying those most in need of urgent intervention.

The practical application of these predictive models could lead to quicker and more accurate patient stratification based on mortality risk in patients with diabetes in the ED. Importantly, integrating such models into clinical workflows should aim to support, rather than disrupt, clinical decision-making. Ensuring seamless integration into existing systems will be critical for enhancing patient care without adding complexity to the healthcare process.

However, that one out of three are at risk of dying within 30 days may warrant investigation and to closely monitor that patient. Furthermore, the model is good at identifying individuals who are at low risk of dying, and that could be excluded from more intense investigations, and instead focus resources on the high-risk group. Additional work needs to be applied before a model can be clinically useful.

## Conclusion

The ML prediction model for 30-day mortality in patients with diabetes or hyperglycemia (glucose were > 11 mmol/L) admitted to the ED demonstrated high accuracy for ruling out patients with a low risk of mortality. It also identified patients at high risk, with approximately 30% of those predicted to be at high risk of mortality within 30 days.

In particular, abnormal blood gas values (pH, lactate, base excess, bicarbonate) are strongly associated with unfavorable outcomes. Other key predictors associated with increased mortality include C-reactive protein, older age, triage-category, sodium and potassium levels in blood, and length of stay in the ED .

These findings have important implications for optimizing patient care and resource allocation in the ED for patients with diabetes or hyperglycemia, helping to better stratify patients based on their risk of mortality, and improving clinical outcomes through timely interventions.

## Supplementary Information

Below is the link to the electronic supplementary material.

Supplementary Material 1.

## Data Availability

The technical appendix, statistical code, and datasets generated and/or analysed during the current study are available from the corresponding author upon reasonable request.
