# Supplementary material for: Development and evaluation of a machine learning prediction model for short-term mortality in patients with diabetes or hyperglycemia at emergency department admission
Source: Cardiovasc Diabetol. 2025 Oct 3;24:383. doi: 10.1186/s12933-025-02954-8 (PMC12492943; doi:10.1186/s12933-025-02954-8)

Supplementary Figure 1


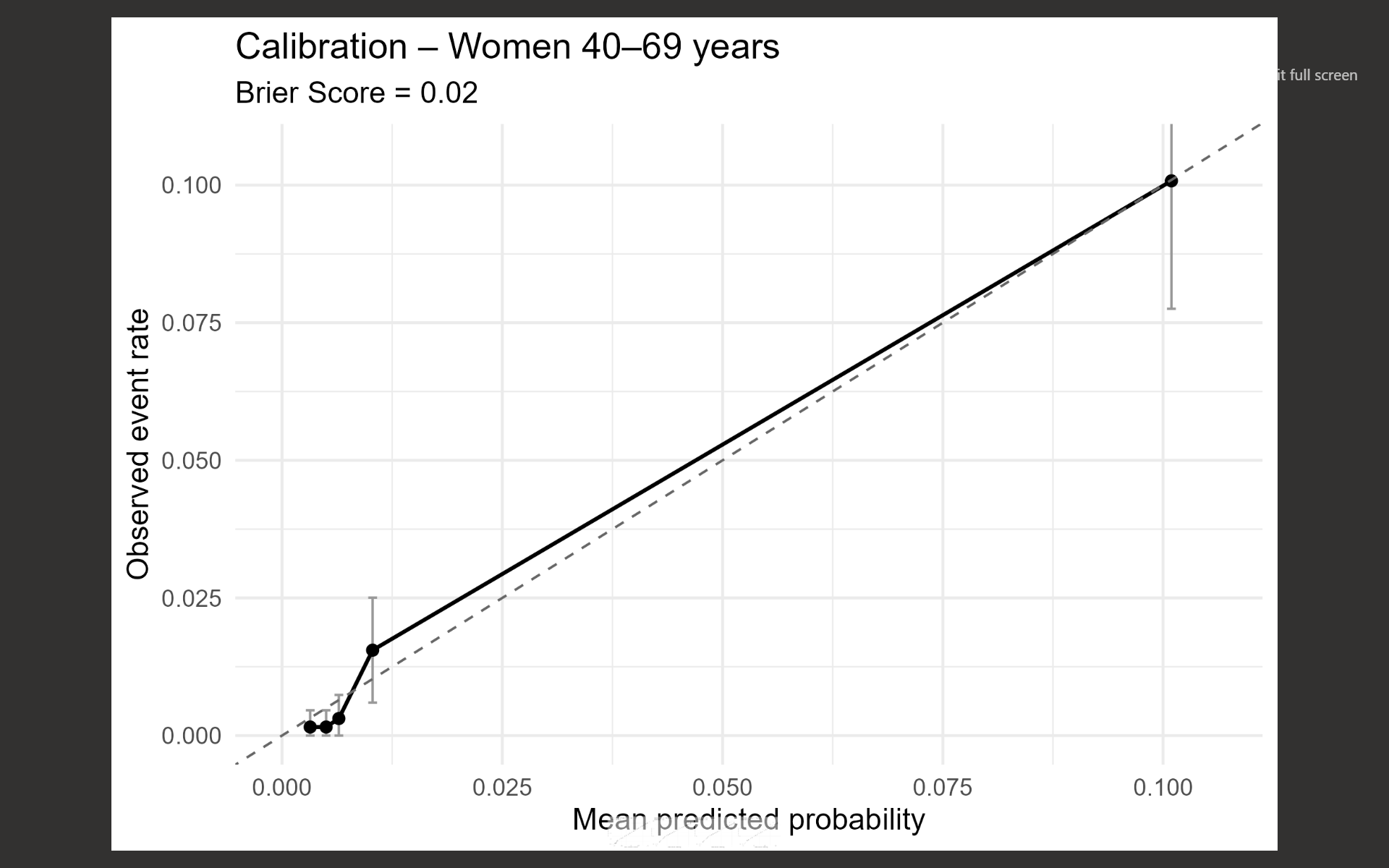


Supplementary Figure 2


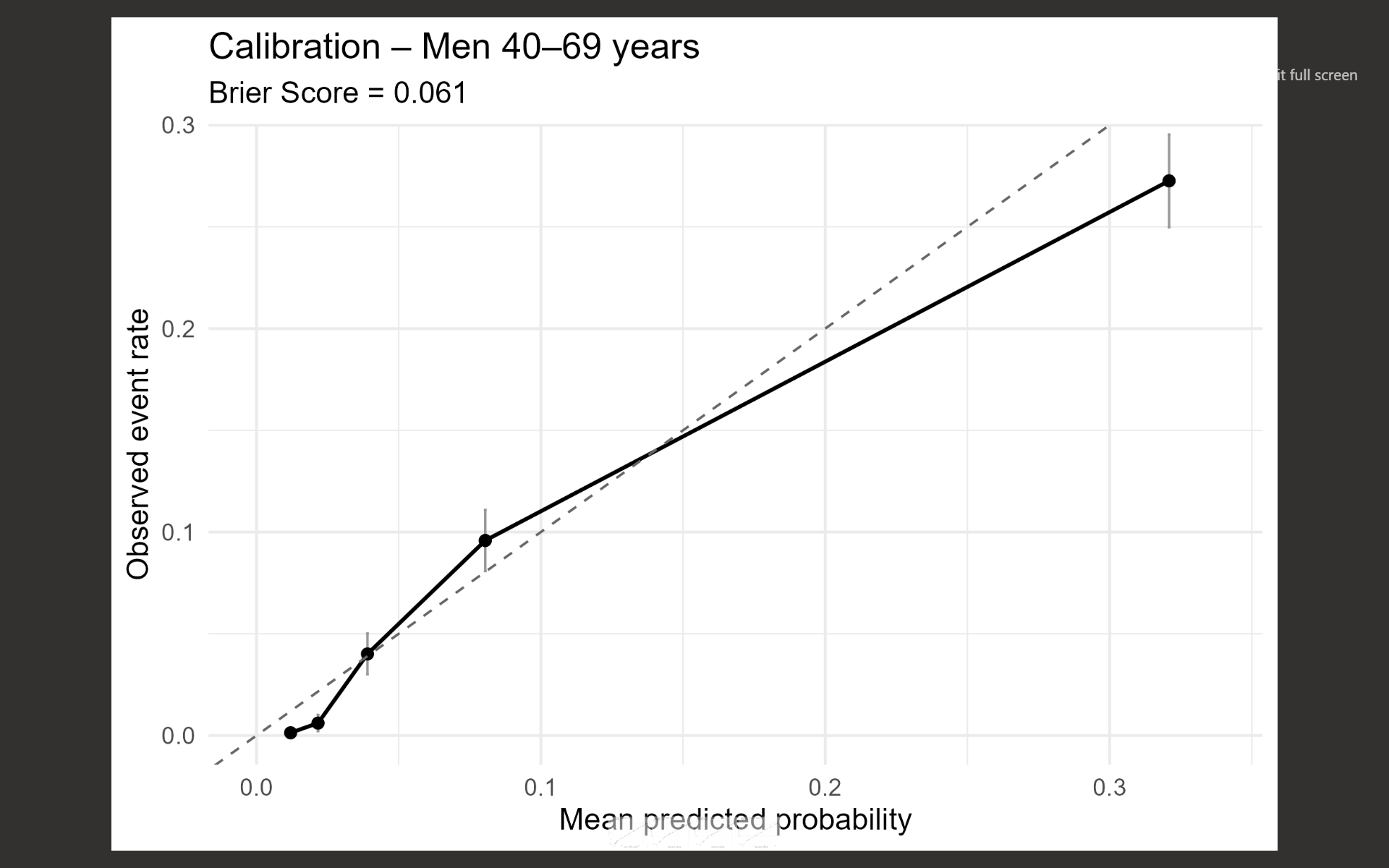


Supplementary Figure 3


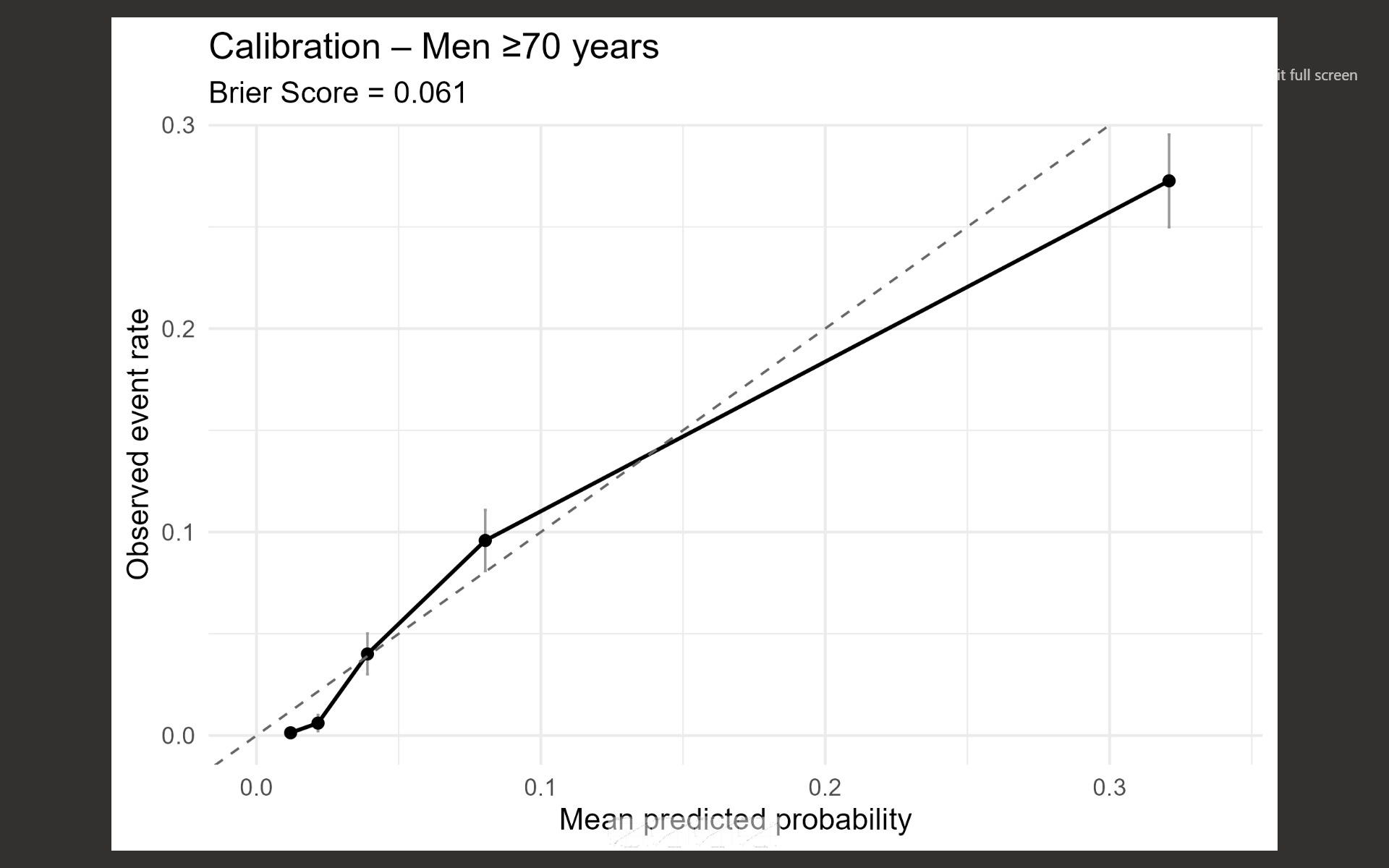


Supplementary Figure 4


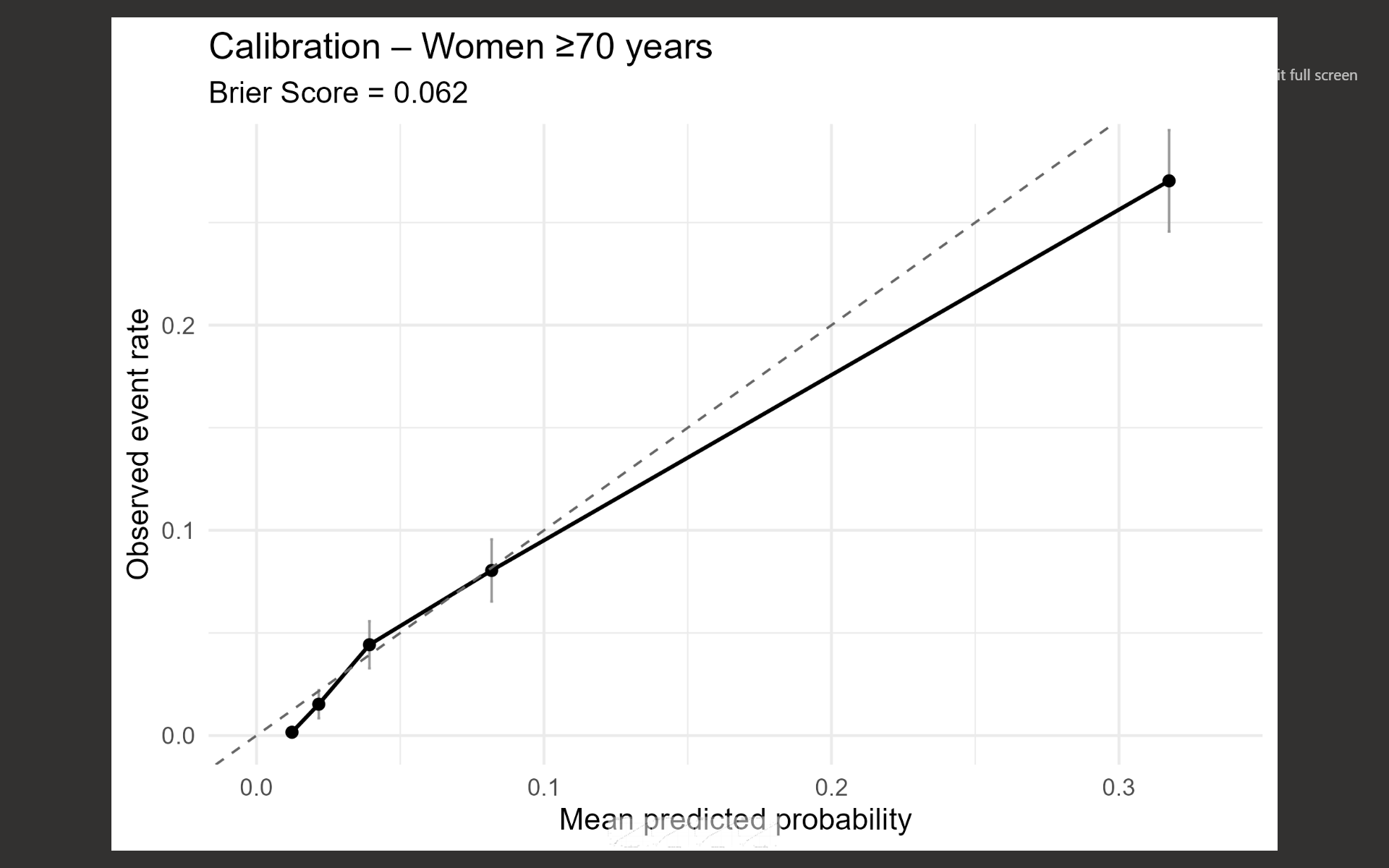


Supplementary Figure 5. Top variables in men 40 to 69 years.


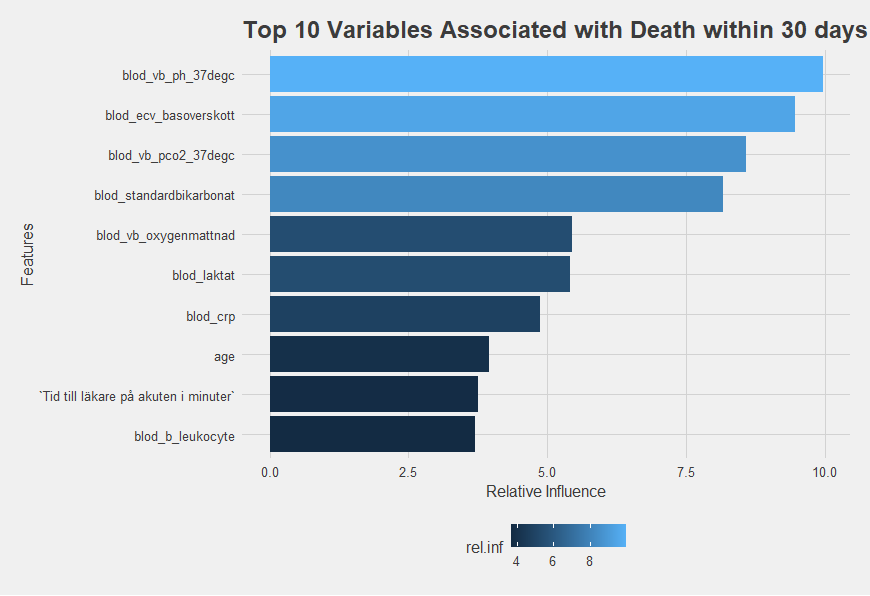


Supplementary Figure 6. Top variables in men ≥70 years.


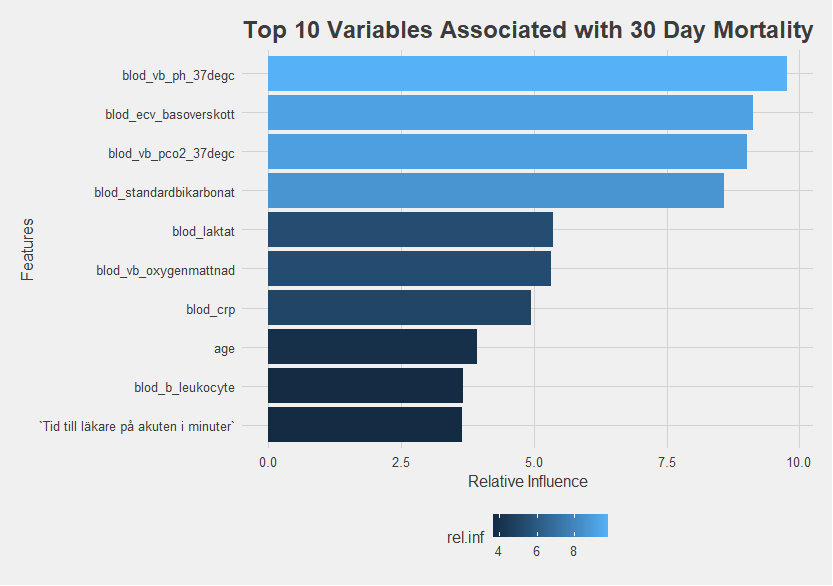


Supplementary Figure 7. Top variables in women 40 to 69 years.


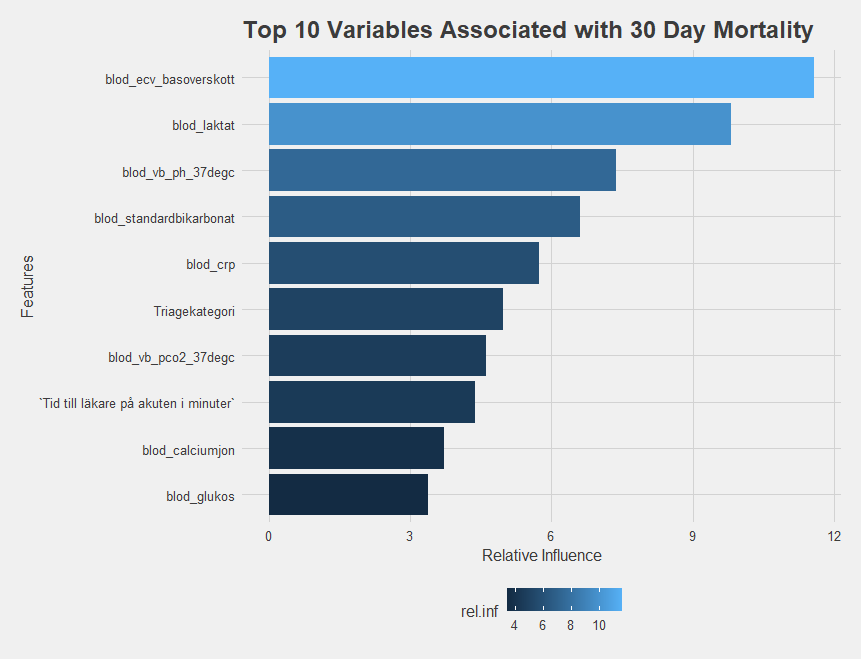


Supplementary Figure 8. Top variables in women ≥70 years.


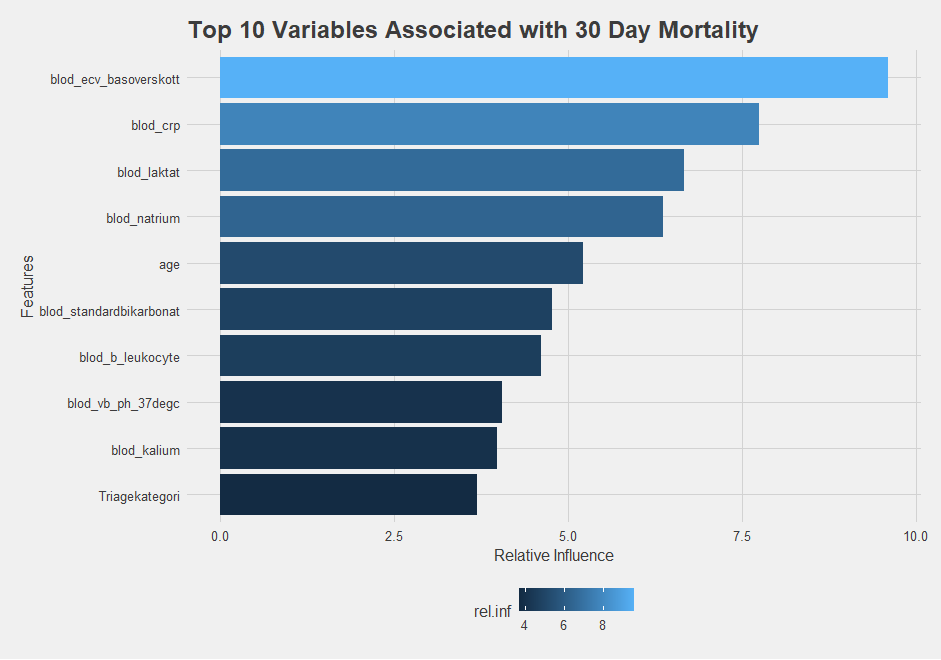

Supplement: Supplementary file 1 — Supplementary Material 1. [file 12933_2025_2954_MOESM1_ESM.docx]
